# A Case Report of Pacemaker-Induced Cardiomyopathy in a Patient With Post-atrioventricular Node Ablation for Atrial Fibrillation

**DOI:** 10.7759/cureus.33930

**Published:** 2023-01-18

**Authors:** Zahid Khan, Tom Rayner, Dinesh Sethumadhavan, Sithu Kyaw

**Affiliations:** 1 Acute Medicine, Mid and South Essex NHS Foundation Trust, Southend on Sea, GBR; 2 Cardiology, Bart’s Heart UK, London, GBR; 3 Cardiology and General Medicine, Barking, Havering and Redbridge University Hospitals NHS Trust, London, GBR; 4 Cardiology, Royal Free Hospital, London, GBR; 5 Cardiology, Barts Heart Centre, London, GBR

**Keywords:** pacemaker-induced cardiomyopathy, shortness of breath (sob), rheumatoid arthritis, comorbid obesity, obstructive sleep apnoea, heart failure with reduced ejection fraction, cardiac resynchronization therapy (crt), progressive interstitial lung disease, severe left ventricular systolic dysfunction, pacemaker complication

## Abstract

Pacemaker-induced cardiomyopathy (PICM) is a rare but well-recognised phenomenon in patients requiring right ventricular (RV) pacing. It can be caused by single-chamber or dual-chamber pacemakers. We present a case of a 64-year-old female patient presenting to the pacemaker clinic with worsening shortness of breath and legs swelling. She was found to have atrial fibrillation and underwent atrioventricular node ablation followed by a dual chamber permanent pacemaker (PPM) implantation as part of a ‘pace and ablate’ strategy to treat refractory symptomatic atrial tachycardia, and the patient was entirely dependent on RV pacing. In the immediate two months following PPM implantation, the patient was seen in the clinic and reported shortness of breath that was attributed to interstitial lung disease. However, a month later her symptoms worsened, stimulating a referral for echocardiography, which demonstrated a fall in her left ventricular ejection fraction (LVEF) from 60% to 30% in just four months after the device implantation. The patient was diagnosed with PICM. The patient’s prognostic heart failure treatment was optimised and her device was upgraded to a cardiac resynchronisation (CRT) device with pacing functionality in an attempt to improve biventricular synchrony. The patient’s symptoms have improved significantly since and a repeat echocardiogram 2 months later showed significant improvement in LVEF to 45-50%.

## Introduction

The number of patients living with permanent pacemakers (PPM) has risen rapidly, with one study finding prevalence trebling between 1995 and 2009 [1]. There is a diverse array of indications for PPM [2], and some patients require a much greater burden of right ventricular (RV) pacing (for example, those high-grade atrioventricular (AV) blocks). With improvements in the management of refractory atrial tachycardia with AV node ablation and an ageing population who are most vulnerable to conduction disease [1], the burden of RV-dependent PPM will likely continue to rise.

A well-studied phenomenon, PICM is frequently characterised by a drop in left ventricular ejection fraction (LVEF) in the setting of high-burden RV pacing (though definitions vary widely and are thought to occur due to interventricular and intraventricular electrical and mechanical dyssynchrony) [3-5]. Patients with frequent RV pacing and paced QRS complexes ≥150 milliseconds should be screened by echocardiogram to assess for PICM. With some estimating the prevalence of this condition to be 10-15% in patients with high RV pacing dependency [6,7], we feel this condition should be a recognised entity and be on the list of differentials for any emergency presentation of paced patients by medical and emergency physicians. We hereby present a case of pacemaker-induced cardiomyopathy (PICM) in a 64-year-old patient.

## Case presentation

**Figure 1:**
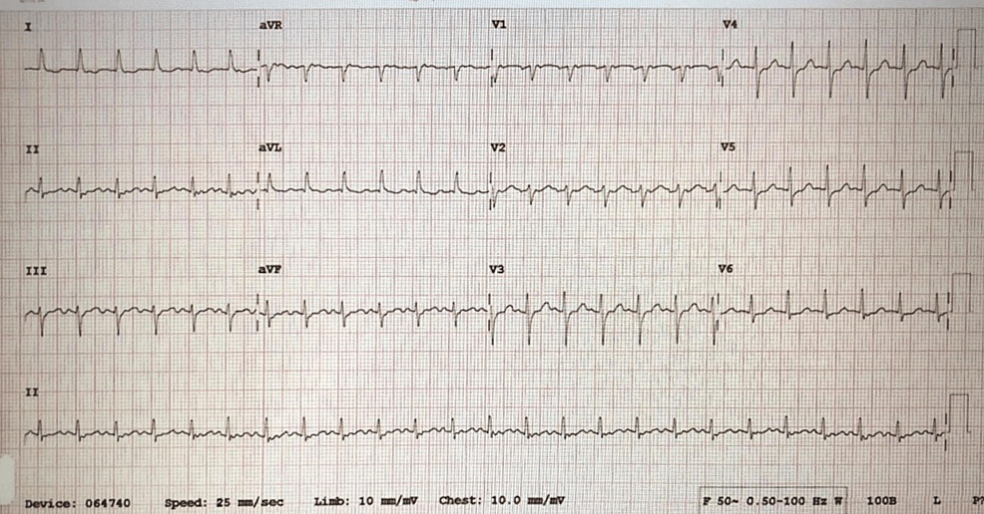
Electrocardiogram shows atrial fibrillation with rapid ventricular response

A 64-year-old female with a background of rheumatoid arthritis, interstitial lung disease (ILD), obesity and obstructive sleep apnoea (OSA) had initially presented to cardiology services with symptomatic paroxysmal atrial fibrillation (PAF) and intermittent typical atrial flutter, refractory to first-line medication in September 2021. Regular medications include verapamil 240 mg once daily, amiodarone 200 mg once daily, digoxin 125 mcg once daily, allopurinol 100 mg twice daily, atorvastatin 20 mg once daily, calcium carbonate/cholecalciferol tablets twice daily, carbocysteine 750 mg capsules three time daily, furosemide 40 mg once daily, lisinopril 10 mg once daily, omeprazole 40 mg once daily, hydroxychloroquine 400 mg once daily, Fostair inhaler and prednisolone 5 mg once daily. Her heart was structurally normal on the echocardiogram, and LVEF was 60%. She underwent two acutely successful cryoablation pulmonary vein isolation and cavotricuspid isthmus (CTI) line ablation procedures in October 2021 and February 2022, both hampered by the early recurrence of palpitations impacting her quality of life. Two months later, in April 2022, the patient attended her local accident and emergency with symptomatic atrial tachycardia with rapid ventricular response despite being on verapamil, amiodarone, and digoxin, which the treating team were unable to chemically cardiovert or control (Figure 1).

**Figure 2:**
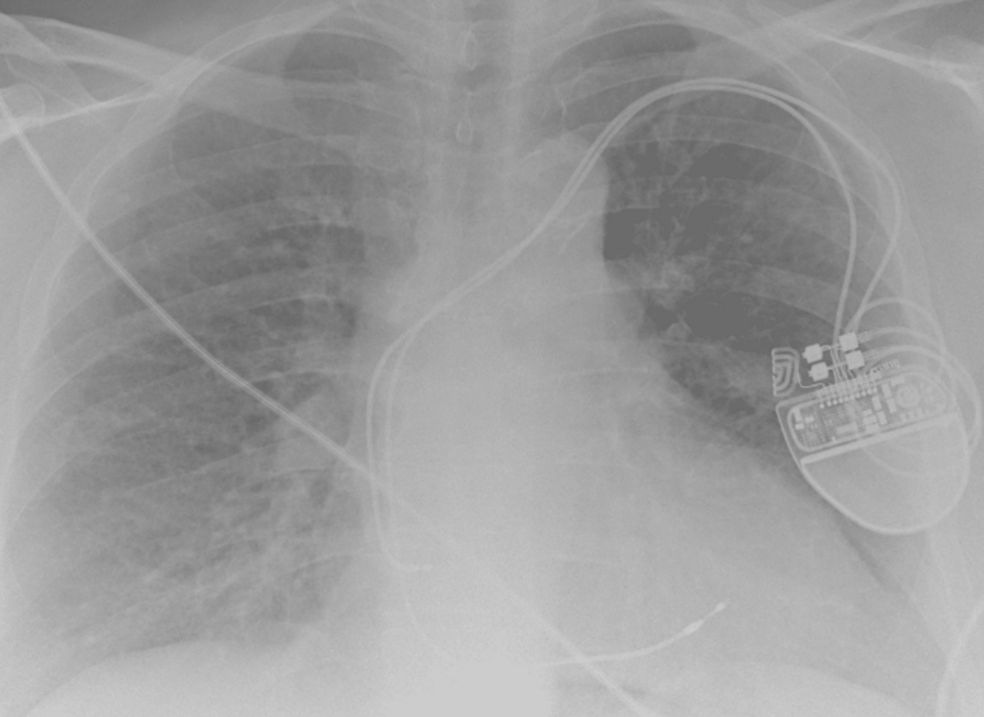
Chest radiography shows dual chamber pacemaker in situ

She was therefore referred to her tertiary electrophysiology service, and she underwent a successful atrioventricular node (AVN) ablation and dual chamber PPM implantation in May 2022 (‘pace and ablate’), with one electrode placed in the right atrium and one in the right ventricle under general anaesthesia. The AVN was identified, and ablation was performed through an F-curve irrigated ablation catheter. Completed heart block (CHB) was achieved with a slow junctional escape rhythm and recovery of rhythm was not noticed after waiting for 15 minutes, hence PPM was implanted. The device was set to DDD mode, with atrial sensing and ventricular pacing and the backup rate was set to 80 beats per minute (bpm). The PPM leads (right atrial (RA) and RV) were active leads, with the RA lead positioned in the RA appendage and the RV lead in the mid-septum. The leads demonstrated stable trends throughout the follow-up period, with no change in sensitivity or impedance. The RV was paced 100% of the time. A post-PPM implant chest X-ray confirmed satisfactory lead positioning when compared to fluoroscopic images during the PPM insertion (Figure 2).

Antiarrhythmic medications including amiodarone, verapamil, and digoxin were stopped and the patient was commenced on bisoprolol 5 mg once daily. The patient was seen in the clinic two months later, reporting shortness of breath starting in the immediate period following PPM implantation. A device check was satisfactory, and the leads demonstrated stable trends. She also reported palpitations, but with a low burden of AF (14%), this was felt to be secondary to normal pacemaker tracking of sinus node beats (max tracking rate of 130 beats per minute). In no instance were there reports of ischaemic chest pain. An echocardiogram was eventually arranged, demonstrating an LVEF of 30%, a 50% reduction in the 4 months following PPM implantation; the patient was significantly short of breath and was diagnosed with PICM (Videos 1-3). Computerized tomography coronary angiogram did not show any obvious coronary artery disease.

**Video 1:** Parasternal long-axis view showing impaired left ventricular systolic function

**Video 2:** Apical four chambers view shows severely impaired left ventricular systolic function

**Video 3:** Parasternal short-axis view shows severely impaired left ventricular systolic function

**Figure 3:**
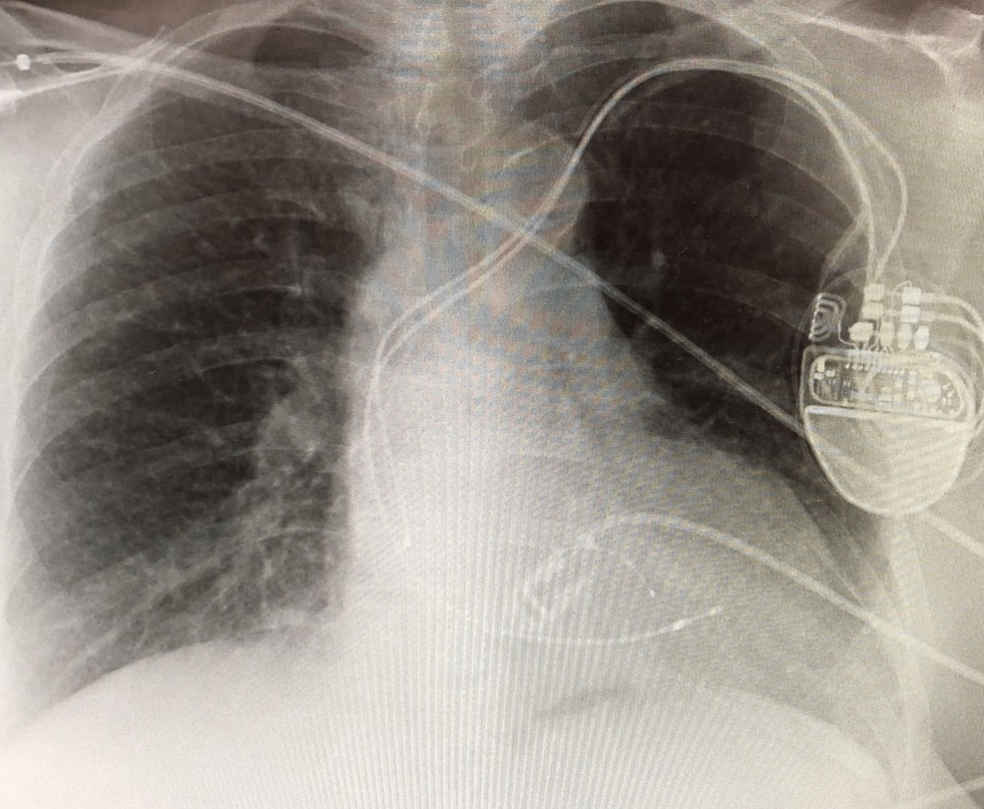
Chest radiograph showing CRT-P in situ CRT-P: cardiac resynchronization therapy pacemaker

Heart failure prognostic medication was optimized and the patient was commenced diuretics and dapagliflozin 3 months prior to the upgradation of the device. Her device was upgraded to a cardiac resynchronization therapy pacemaker (CRT-P) device with a pacing function in November 2022 with the introduction of a left ventricular lead via the coronary sinus (Figure 3 and Video 4). The post-procedure QRS duration was 118 ms, an indication of adequate biventricular synchrony. Her latest device check showed a total burden of AF 14%, longest episode of 5 and half hours and a mean heart rate of 70 bpm. Repeat echocardiogram 2 months later showed improved LVEF 45-50%. 

**Video 4:** Cardiac resynchronization therapy pacemaker video with three leads including left ventricular lead

## Discussion

We present the case of a patient in her 60s with a diagnosis of PICM with high RV pacing dependency without ischaemic heart disease and a structurally normal heart prior to PPM insertion. Definitions vary widely, but in general, PICM is seen as a fall in LVEF in the context of high-burden RV pacing. This definition may result in inadequate capture of patients with PICM, and studies have shown that patients may suffer heart failure symptoms with reasonably preserved LVEF [3,8]. As in our case, and in similar reports [9], patients often present with features of left ventricular failure in the context of recent PPM insertion, though a single study demonstrated that a significant number of patients with PICM had no clinical evidence of heart failure [7]. PICM is thought to be caused by electrical and mechanical dyssynchrony resulting from frequent RV pacing [4,5]. RV pacing may contribute to structural changes within the left ventricle (LV) and left atrium (LA) that contribute to the deterioration in LVEF observed in PICM [10,11]. It can be difficult to delineate whether changes in LVEF can be wholly attributed to PICM, as the population at risk of PICM often suffer from pathologies known to cause deleterious effects on LV structure (e.g., ischaemic heart disease). Therefore, any patient presenting with reduced LVEF, and RV pacing should have other causes thoroughly investigated as part of their initial workup.

The risk of PICM is thought to be greatest in male and older patients, those with increased native QRS duration, greater QRS duration following PPM insertion, a greater burden of RV pacing, and pre-PPM reduced LVEF [12]. Studies suggest PICM occurs within 12 months after PPM insertion [4,6]. Bansal and colleagues report an incidence of 13.8% PICM in their cohort when followed up over 14.5 months [4]. This is substantiated by Yu et al., finding that 9% of their RV-paced cohort developed PICM over a 12-month follow-up period [6]. However, beyond the 12-month follow-up period, there is some evidence that the incidence of PICM may plateau, with Dreger and colleagues reporting only marginally greater PiCMP cases (15.4%) over a 15-year follow-up period for RV-paced individuals with structurally normal hearts [5]. There is no consensus regarding the timing of LVEF assessment post RV PPM placement but based on the modest evidence supporting the early development of PICM, some have recommended echocardiography at 12 months [5].

As in our case, patients with PICM should have prognostic medications instituted and acute complications, such as pulmonary oedema managed appropriately [13]. Cardiac resynchronisation therapy (CRT) has become a common method for the management of PICM, principally aiming to improve mechanical dyssynchrony, and there is evidence to suggest it may in part improve LVEF [14]. There is data to suggest that biventricular pacing is associated with improvements in LV performance and geometry, as well as on functional status [15]. The question as to whether patients with high-burden RV pacing CRT from the outset should have, rather than upgrade to biventricular pacing in the event of PICM is unanswered, and most studies have focused on device upgradation to CRT [16]. The latest data suggest that conduction system pacing may provide an opportunity to prevent PICM from occurring in the first place. Sanchez, et al. suggested that leadless RV in patients requiring pacing may reduce the risk of infections, lead failures and pneumothoraces [16, 17]. However, more research is needed to verify the findings of this study about leadless pacemakers.

## Conclusions

PICM is an infrequent but important complication of RV pacing and should be readily thought of when assessing any patient in the acute setting with symptoms of heart failure. This is particularly true when the patient presents soon after PPM insertion. Investigation of any patient with these symptoms should be investigated with echocardiography, and medical management of heart failure and its complications instituted. The patient should be referred for cardiology assessment and a potential upgrade to biventricular pacing.
